# Anterior Thalamic High Frequency Band Activity Is Coupled with Theta Oscillations at Rest

**DOI:** 10.3389/fnhum.2017.00358

**Published:** 2017-07-20

**Authors:** Catherine M. Sweeney-Reed, Tino Zaehle, Jürgen Voges, Friedhelm C. Schmitt, Lars Buentjen, Viola Borchardt, Martin Walter, Hermann Hinrichs, Hans-Jochen Heinze, Michael D. Rugg, Robert T. Knight

**Affiliations:** ^1^Departments of Neurology and Stereotactic Neurosurgery, Otto von Guericke University Magdeburg, Germany; ^2^Department of Behavioral Neurology, Leibniz Institute for Neurobiology Magdeburg, Germany; ^3^Department of Psychiatry, Eberhard Karls University Tübingen, Germany; ^4^German Centre for Neurodegenerative Diseases (DZNE), Otto von Guericke University Magdeburg, Germany; ^5^Center for Vital Longevity and School of Behavioral and Brain Sciences, University of Texas Dallas, TX, United States; ^6^Helen Wills Neuroscience Institute and Department of Psychology, University of California, Berkeley Berkeley, CA, United States

**Keywords:** cross-frequency coupling, phase amplitude coupling, anterior thalamic nucleus, theta, gamma, high frequency band, resting state, intracranial EEG

## Abstract

Cross-frequency coupling (CFC) between slow and fast brain rhythms, in the form of phase–amplitude coupling (PAC), is proposed to enable the coordination of neural oscillatory activity required for cognitive processing. PAC has been identified in the neocortex and mesial temporal regions, varying according to the cognitive task being performed and also at rest. PAC has also been observed in the anterior thalamic nucleus (ATN) during memory processing. The thalamus is active during the resting state and has been proposed to be involved in switching between task-free cognitive states such as rest, in which attention is internally-focused, and externally-focused cognitive states, in which an individual engages with environmental stimuli. It is unknown whether PAC is an ongoing phenomenon during the resting state in the ATN, which is modulated during different cognitive states, or whether it only arises during the performance of specific tasks. We analyzed electrophysiological recordings of ATN activity during rest from seven patients who received thalamic electrodes implanted for treatment of pharmacoresistant focal epilepsy. PAC was identified between theta (4–6 Hz) phase and high frequency band (80–150 Hz) amplitude during rest in all seven patients, which diminished during engagement in tasks involving an external focus of attention. The findings are consistent with the proposal that theta–gamma coupling in the ATN is an ongoing phenomenon, which is modulated by task performance.

## Introduction

Neuronal activity oscillates at multiple frequency ranges, transiently forming synchronous networks associated with cognitive processing (Llinas, 1988; Hutcheon and Yarom, 2000; Varela et al., 2001; Buzsáki and Draguhn, 2004). Synchrony between neural oscillators occurs not only within a given frequency range but also between frequency bands (Tass et al., 1998; Canolty and Knight, 2010), referred to as cross-frequency coupling (CFC). In the last decade, the modulation of fast oscillations in the gamma frequency range (>30 Hz) by slower frequency oscillations in the theta range (4–8 Hz) has been identified in several brain structures, including the neocortex (Canolty et al., 2006), the hippocampus (Axmacher et al., 2010) and the anterior thalamic nucleus (ATN; Sweeney-Reed et al., 2014). Low frequency oscillations (LFOs), which includes those in the theta range, are modulated by the external environment via sensory and motor inputs and by endogenous cognitive processes (Schroeder and Lakatos, 2009; Canolty and Knight, 2010). Slower rhythms enable long-range communication between remote brain areas, with their phase reflecting local neuronal excitability (Canolty and Knight, 2010), while high frequency activity (high frequency band; HFB, 60–200 Hz) is believed to underlie information processing in localized cortical regions (Canolty et al., 2006; Jensen et al., 2007). On the basis of previously identified functional correlations and the plausibility of the postulated physiological mechanisms involved (Canolty and Knight, 2010), we focus here specifically on phase–amplitude coupling (PAC), although we note that roles for other forms of CFC, such as phase–phase and amplitude–amplitude coupling, have also been proposed (Canolty and Knight, 2010). Strong theta–HFB PAC has been hypothesized to induce long-term synaptic potentiation (LTP) regulating synaptic strength, on the basis of induction of LTP using high gamma bursts at a theta interval and also of strong correlations between PAC and learning and between learning and synaptic LTP (Axmacher et al., 2006; Canolty and Knight, 2010). To further examine the role of thalamic PAC in cognitive processing, we investigated whether thalamic PAC is a continuous phenomenon, modulated by external events, or whether it arises only at specific points in time to support goal-related cognitive processes.

The resting state is defined as an awake and alert state in which the mind is wandering, but the participant is not engaged in any specific cognitive task. It is an internally-focused cognitive state, involving spontaneous, stimulus-independent cognition, or internally-cued thoughts, while individuals are not focused on the external environment (Greicius et al., 2003; Buckner et al., 2008; Carhart-Harris and Friston, 2010). In contrast, a cognitive state involving an external focus of attention means an individual is engaged in a cognitive task involving stimuli or cues from the environment (Buckner et al., 2008; Carhart-Harris and Friston, 2010). Brain areas have the ability to form intrinsic functional connectivity networks, which are characterized by temporally synchronized patterns of activation (Cohen et al., 2008). Functionally connected brain structures that are more active during the resting state than during tasks requiring attention to external events comprise the default mode network (DMN; Shulman et al., 1997; Raichle et al., 2001; Greicius et al., 2003; Raichle, 2015). During the resting state, the DMN—which has been implicated in self-referential and memory-based processing (Vatansever et al., 2015)—is active. Although its functional role remains speculative (Broyd et al., 2009; Raichle, 2015), the DMN is thought to support the “free-wheeling” nature of ongoing cognitive activity during rest (Leech and Sharp, 2014). It has also recently been suggested that it functions as a global integrator of information (van den Heuvel and Sporns, 2011; Vatansever et al., 2015). Imaging studies indicate that the thalamus modulates resting cerebral activity (De Luca et al., 2006; Mantini et al., 2007), with damage to the anterior thalamus disrupting the DMN and leading to global cognitive deficits (Jones et al., 2011).

We predicted PAC in the ATN during rest for the following reasons. First, our hypothesis focused on the ATN, because lesion evidence suggests functional connectivity between the ATN and the DMN (Jones et al., 2011), suggesting a role for the ATN during rest. Second, we predicted PAC at rest, because theta–gamma PAC has been observed at rest in the neocortex in electrocorticographic (ECoG; Foster and Parvizi, 2012) and in magnetoencephalographic (MEG; Florin and Baillet, 2015) recordings. Further, with regard to the thalamus, intrinsic as well as network thalamic oscillations are influenced by neocortical oscillations, with rhythmic thalamic and cortical spike bursts occurring during rest modulating neocortical plasticity (Steriade, 2001). Third, considering a potential role for the thalamus during rest, it has been proposed, on the basis of neuroimaging studies, that the cortico–cortical interactions underlying switching between a resting and an externally-focused state may be mediated by the thalamus (Greicius et al., 2003; Di et al., 2013; Wang et al., 2014).

PAC provides a potential mechanism for two reasons. First, PAC has been identified throughout the cortex during the resting state. In particular, PAC in MEG recordings was correlated between reconstructed sources that corresponded with nodes of the DMN identified on fMRI (Florin and Baillet, 2015), suggesting that PAC could contribute to maintaining this network. A mechanism has been proposed, termed the synchronized gating hypothesis, by which PAC could underpin dynamic and flexible network formation involving subcortical as well as cortical hubs (Florin and Baillet, 2015). PAC in the ATN could provide a mechanism by which the ATN fulfills a role as a hub, coordinating and switching between different networks. It has been hypothesized that PAC facilitates neural assembly selection (Yanagisawa et al., 2012; Sweeney-Reed et al., 2014), and task-switching would involve a change in the neural assemblies which are coordinated. Second, we previously observed differing gamma frequency range involvement in PAC in the ATN according to memory formation success (Sweeney-Reed et al., 2014), which is consistent with the notion that different tasks involve coordination of different neural assemblies firing at particular gamma range frequencies.

PAC has been found to be sensitive to nonsinusoidal properties of the LFO (Aru et al., 2015; Jensen et al., 2016; Cole and Voytek, 2017; Cole et al., 2017; Vaz et al., 2017). The shape of the waveform varies with the frequency and brain location examined (Cole et al., 2017; Vaz et al., 2017). A sawtooth LFO waveform, as found in motor cortical beta oscillations, can result in PAC which almost completely correlates with the degree of waveform symmetry, as measured by the degree of sharpness in peaks compared with troughs, or the ratio between peak and trough sharpness (Cole et al., 2017). It has been suggested, on the basis of modeling, that waves with such a sharp form could result from synchronous synaptic activity (Cole et al., 2017). We investigated this possibility in the resting thalamic theta–HFB PAC.

We hypothesized that PAC in the ATN reflects not only externally-focused cognitive processing, as previously identified during a task involving memory formation for presented images (Sweeney-Reed et al., 2014), but is also present when participants are at rest. We postulated that the PAC taking place during tasks in which participants engage with external stimuli differs from that underlying unstructured, internally-focused cognitive processing, because such tasks repeatedly and robustly engage specific neural assemblies each time the task is performed. In contrast, during rest, the neural assemblies, which form and dissolve over time, are likely to involve more diverse brain regions and oscillatory frequencies, because a broader range of cognitive and affective acts is taking place.

## Materials and Methods

Intracranial electrophysiological data were recorded directly from the ATN and dorsomedial thalamic nuclei (DMTN) from seven patients with electrodes implanted for the treatment of pharmacoresistant focal epilepsy. This study was carried out in accordance with the recommendations of the Local Ethics Committee of the Otto-von-Guericke University, Magdeburg. All patients gave written informed consent in accordance with the Declaration of Helsinki. Detailed clinical and neuropsychological evaluations are available in other publications (Sweeney-Reed et al., 2014, 2016a).

### Electrophysiological Data

The electrophysiological data were recorded at the patients’ bedside 3–5 days after electrode implantation, with the head end of the bed raised supporting a relaxed sitting position. During recording of the resting state data, the patients were instructed to remain awake, with their eyes open, and ambient noise and external distraction were minimized. Next, data were recorded during the cognitive paradigms involving external stimuli. Resting data length varied between 2 min and 5 min, due to the limited total time available for electrophysiological data recording before the electrode probes were linked to the stimulation device and no longer available for data recording. The maximal continuous data length in which no artifacts were visible was selected for each patient for each recording condition (during rest and during two tasks involving external stimuli). We analyzed continuous data, instead of removing sections containing artifacts, to avoid introducing altered waveform shapes, because nonsinusoidal waveform shapes could affect PAC values (Cole and Voytek, 2017). A minimum of 30 s was used (Cole et al., 2017). Five additional episodes with a minimum length of 30 s were identified and analyzed separately (see Supplementary Material). The temporal resolution of intracranial electrophysiological recordings and the millisecond scale of cognitive acts (Varela et al., 2001) assure that the data length provided a time period sufficient for reliable PAC and representative of the internally-focused cognitive processing under investigation, and a buffer was used to maximize the available data at the lower frequencies during wavelet transformation. A 50 Hz notch filter was applied to all data to remove line noise using EEGLAB, Version 13.4.4b (Delorme and Makeig, 2004).

Resting state data were recorded from all seven patients, and data were recorded from six of the patients during each of the two cognitive tasks requiring external attentional focus. In one task, participants viewed a series of photographic scenes, which they judged as indoors or outdoors via a button press with the left or right (counter-balanced across patients) index finger. Analysis of these data has been previously reported in a memory encoding study, in which the data were epoched for each stimulus and categorized according to whether memory formation was successful by reference to a subsequent memory test (Sweeney-Reed et al., 2014). A practice session, in which both the encoding and retrieval stages were performed, rendered memory encoding intentional. The data evaluated here were recorded during the memory encoding phase of the main experiment. The other task was a novelty oddball paradigm, in which participants viewed outdoor scenes and responded via button press with the right index finger to infrequently presented target oddball stimuli (Zaehle et al., 2013). The recognition test practice run was performed after completion of the oddball task blocks, rendering the memory aspect of the oddball task unintentional. In the present study, the first 4.5–5 min of continuous, artifact-free data (depending on clean data length available) from these two cognitive tasks were analyzed, so that data length was similar to the resting state data, in order to enable a comparison between internally-focused cognitive processing and processing involving an external focus.

### Electrode Localization

**Figure 1:**
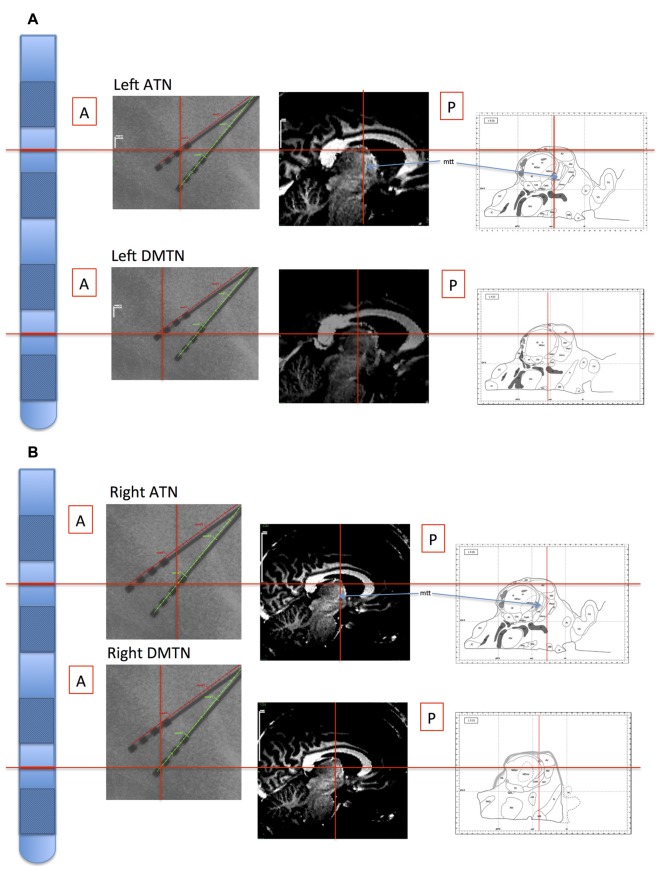
Illustration of location of recording sites in Patient 4. The electrode probes are represented on the far left, and the levels of the inter-contact points, corresponding with the bipolar channel for each thalamic nucleus, are indicated with horizontal red lines. Left panels: the intra-operative stereotactic X-ray images (lateral view) from which location coordinates were derived. Middle panels: the corresponding locations are superimposed on the pre-operative structural MRI images. By co-registering the images with post-operative CT images, the coordinate positions were determined with reference to atlases (Schaltenbrand and Wahren, 1977; Morel, 2007; Buentjen et al., 2014). Right panels: channel locations with reference to the Morel atlas. (Line drawings from Morel (2007): Copyright © 2007. From “Stereotactic Atlas of the Human Thalamus and Basal Ganglia” by A. Morel. Reproduced by permission of Taylor and Francis Group, LLC, a division of Informa plc). **(A)** Left channels. **(B)** Right channels. ATN, anterior thalamic nucleus; DMTN, dorsomedial thalamic nucleus; mtt, mammillothalamic tract.

The stereotactic target structure was the ATN, and electrode contacts (quadripolar brain electrodes from Medtronic, Minneapolis, MN, USA: model 3387) were additionally located in the DMTN of six patients, because deeper electrode insertion enhances stability of the target ATN electrode probe. Intra-operative x-ray and post-operative CT images were co-registered with the pre-operative structural magnetic resonance imaging scans used to plan electrode placement (Figure 1). Details regarding electrode localization are available (Buentjen et al., 2014; Sweeney-Reed et al., 2014, 2016b). Electrophysiological data were recorded at a sampling frequency of 512 Hz from eight intracranial contacts (four each side) using a Walter Graphthek amplifier against a nose reference. The data were subsequently re-referenced offline to yield a bipolar montage. A left and a right ATN channel were obtained for each of the seven patients, along with bilateral DMTN channels for six patients.

### Power

The power spectral densities of the resting data were calculated for each patient using Welch’s average modified periodogram as implemented in EEGLAB, dividing the data into eight sections with 50% overlap and windowing each section with a Hamming window (Delorme and Makeig, 2004). The power in the LFO was calculated from 3 Hz to 14 Hz and averaged across patients, in order to investigate whether theta oscillations were present in the data. The high frequency power was calculated from 30 Hz to 200 Hz and smoothed using a 5 Hz moving average.

### Phase–Amplitude Coupling

PAC measures the temporal relationship between the phase of one signal and the amplitude of another signal at specific frequencies, and was calculated using Matlab, modifying the freely available Matlab Toolbox for Estimating PAC (Onslow et al., 2011), implementing a well-established approach (Canolty et al., 2006). The signals were wavelet transformed using a 6-cycle Morlet wavelet, resulting in an analytic representation of the signal *Z(t)*:

(1) $$Z(t)=A(t)\cdot e^{i\theta (t)}$$

where *A(t)* is the amplitude envelope of the signal, and *θ(t)* is the phase angle. Series of instantaneous amplitude and phase estimates were derived from these complex signals: amplitude is calculated as the absolute value of the signal, and phase is determined by taking the arctangent of the imaginary divided by the real part. The center frequencies used for phase were 2–20 Hz at 2 Hz intervals, and for frequency 30–240 Hz at 5 Hz intervals. Edge-effects occur at the start and end of the wavelet power spectrum (Torrence and Compo, 1998; Sweeney-Reed and Nasuto, 2007). We therefore applied a buffer to each end of the time series before wavelet calculation, which was removed before PAC calculation. New, composite complex signals were generated using the instantaneous phases of the LFOs combined with the envelopes of the high frequency (HFB) amplitudes. The absolute value of the mean of the composite signal provided the modulation index (MI), as follows:

(2) MI=|mean(Z(t))|

An MI greater than zero indicates a tendency for amplitude in the high frequency signal to peak at a particular point in the phase cycle of the low frequency signal (Onslow et al., 2011). A distribution against which to test the statistical significance of the MI for each channel was generated by dividing the signal into two sections and swapping amplitude and phase blocks in order to preserve the statistical properties of the data in the surrogates (Canolty et al., 2006; Foster and Parvizi, 2012; Aru et al., 2015; Gohel et al., 2016).

For each patient, the frequency pattern of significant PAC was defined two-dimensionally, in terms of the frequency of the broad theta (4–8 Hz) phases and that of the broad gamma (33–200 Hz) amplitudes with which they were coupled. First, these patterns were compared in a pairwise fashion between patients using the two-sample two-dimensional Kolmogorov-Smirnov test (2-D KS test; Peacock, 1983), in order to evaluate whether the frequencies of the coupled oscillations were consistent across individuals. To facilitate a direct comparison between internally- and externally–focused conditions, the phase–amplitude frequency combinations, for which PAC was calculated and the significance rendered binary, were summed across patients to compare the resting condition with the conditions requiring external attention, again including frequencies across broad theta (4–8 Hz) and gamma (33–200 Hz) frequency ranges. These coupling patterns were rescaled between 0 and 1, to allow direct comparison between conditions with differing participant numbers. The average theta–gamma PAC was also compared between conditions using a Wilcoxon test. This comparison was made between MI calculated for theta phase over 4–6 Hz and amplitude over 83–148 Hz, based on the present findings and consistent with PAC identified previously in the neocortex (80–150 Hz; Canolty et al., 2006). The number of significant frequency combinations was compared between conditions using a Wilcoxon test. Theta phase distributions were assessed using Kuiper’s test, which is used to compare circular distributions (Kuiper, 1960). Tests were performed both at a group level and also pairwise between participants, with Bonferroni correction for the multiple (21) pairwise comparisons.

### Waveform Shape

We evaluated the waveform symmetry to ascertain whether the shape of the LFO played a role in the PAC. We compared two approaches to obtaining the LFO. In the first, we applied a finite impulse response bandpass filter to obtain the 4–6 Hz band. In the second, we used empirical mode decomposition (EMD), which is an adaptive, data-driven time–frequency decomposition method, in which frequency components, referred to as intrinsic mode functions (IMFs), are obtained using an iterative sifting process (details of the method and its application to EEG signals are provided elsewhere (Huang et al., 1998; Andrade et al., 2006; Sweeney-Reed and Nasuto, 2007). EMD has been suggested for evaluating waveform shape in amplitude–amplitude CFC, because of its applicability to nonlinear, nonstationary signals such as EEG (Yeh et al., 2016). Following decomposition, the IMF whose peak was in the 4–6 Hz range was selected.

The shape of the waveforms was quantified by calculating the ratio between the sharpness of the peaks and the troughs, following the approach by Cole et al. (2017). Briefly, the sharpness of each peak was calculated by averaging the difference between peak voltage and voltage 5 ms before the peak with the difference between the peak voltage and that 5 ms after the peak. Trough sharpness was calculated analogously. A mean value was calculated across all the peaks and across all the troughs respectively, and the sharpness was defined using the ratio between the two. Correlation was then calculated between the sharpness ratio and the degree of PAC, employing the sharpness ratio as derived from bandpass filtering and as obtained following EMD. The degree of PAC was taken as the peak theta–HFB PAC and also as the mean in the ranges 4–6 Hz and 80–150 Hz.

## Results

**Figure 2:**
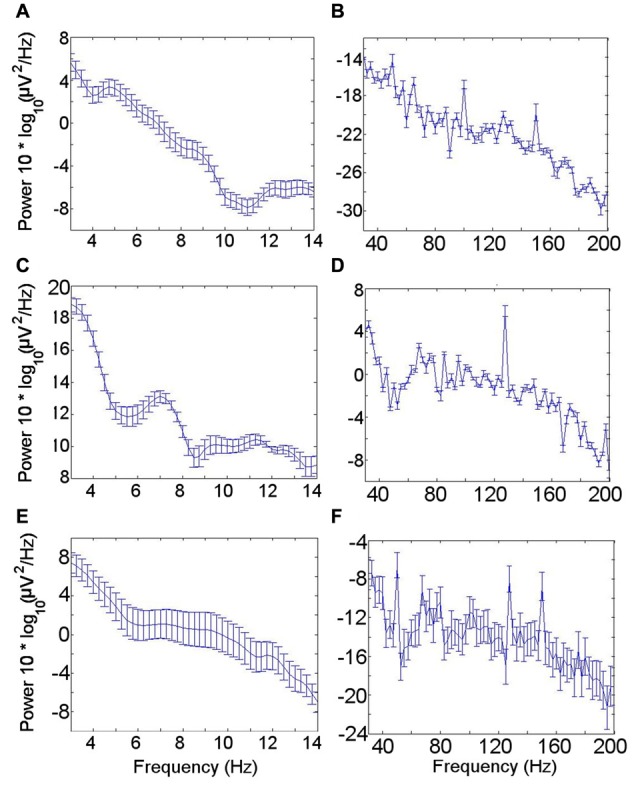
Low frequency oscillation (LFO) and high frequency band (HFB) power spectra. **(A)** LFO during rest. A peak in the theta frequency range is visible in the mean power spectrum across all seven participants during the resting condition. **(B)** HFB during rest. No clear gamma peak corresponding with the gamma frequency range coupled with the theta rhythm was identified. **(C)** LFO during encoding task. **(D)** HFB during encoding task. **(E)** LFO during novelty oddball task. **(F)** HFB during novelty oddball task. Error bars indicate the standard error of the mean across participants.

The mean power spectral density for the LFO across patients during the resting condition showed a peak in the theta oscillation at 4–6 Hz (Figure 2A). A theta peak was seen in upper theta (6–8 Hz) during the memory encoding task (Figure 2C) but not during the novelty oddball paradigm (Figure 2E). Gamma spectral power did not show a clear peak in any of the conditions (Figures 2B,D,F).

**Figure 3:**
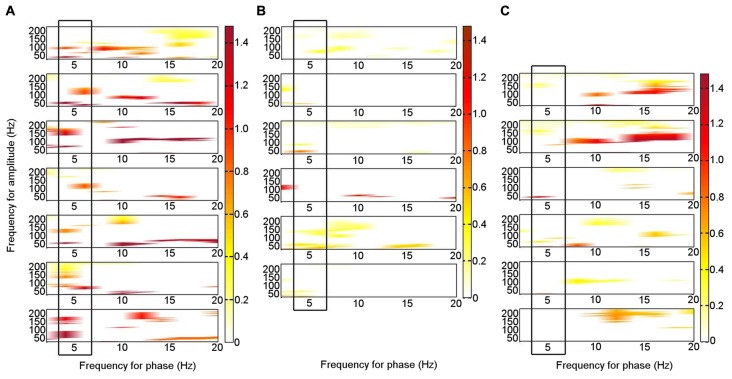
Phase–amplitude coupling (PAC) between theta phase and high frequency band amplitude in the left ATN in seven individual participants. PAC is shown when it was significant (permutation test: criterion *p* < 0.05). The panels are aligned such that PAC from the same patient is shown in each row. The panels are for Patients 1–7, from the top down. **(A)** At rest. **(B)** During memory encoding task. **(C)** During novelty oddball task. The boxes highlight the consistent PAC pattern across participants during rest **(A)**, which is not seen during the externally focused tasks **(B,C)**.

During rest, significant PAC was identified in the left ATN between theta (4–6 Hz) phase and HFB (80–150 Hz) amplitude (permutation test: *p* < 0.05) on an individual level in all seven patients (Figure 3A; Supplementary Figure S1). Similar theta–HFB PAC was also identified in the right ATN in two patients, in the left DMTN in three of the six patients with left DMTN contacts, and in three of the five patients in the right DMTN (one was excluded, because 30 s of artifact-free resting data were not available). The findings that theta–HFB PAC was significant only in two patients in the right ATN, compared with all seven for the left ATN, suggested a lateralization effect. However, on direct comparison, neither the difference between left and right ATN PAC nor that with left or right DMTN was significant (Wilcoxon test: *p* > 0.05). On the basis of the finding of significant PAC in the left ATN on an individual level in all the patients, we focused on the left ATN PAC in our further analyses.

To enable a direct comparison between the patterns of frequencies that were coupled during the resting condition in the seven individual patients, we performed a binary classification of PAC according to whether it was significant or not for each theta frequency range phase combined with each gamma frequency range amplitude for each patient, including broad theta (4–8 Hz) and gamma (33–200 Hz) ranges. A two-sample 2-D KS test was applied pairwise to the patterns of significant PAC between all seven patients (threshold *p* = 0.0024; criterion *q* = 0.05, Bonferroni-corrected for 21 comparisons). In 17 of 21 (81%) comparisons, the theta–gamma PAC did not differ significantly between participants, suggesting a consistent PAC pattern across individuals.

**Figure 4:**
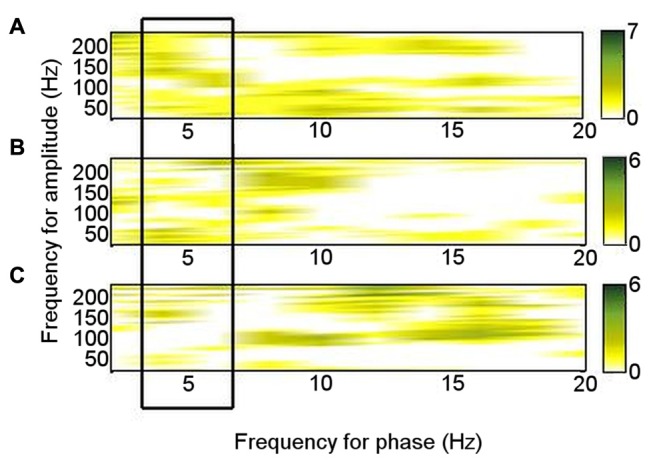
Phase–amplitude coupling (PAC) pattern for each condition. Each frequency–frequency point was given a binary value according to whether PAC was significant in each individual, and these values were summed over the total number of participants who performed each task. **(A)** During rest. **(B)** During encoding task. **(C)** During novelty oddball paradigm.

Of the six patients who also performed the memory encoding task involving judgments of whether scenes were in- or outdoors, Patients 2, 3, 5 and 6 had significant left ATN PAC involving the theta frequency range in which PAC was found to be significant at rest (4–6 Hz), but coupling was with the low gamma range. Only Patients 1 and 5 had significant left ATN PAC involving the HFB range in which PAC was found to be significant at rest (Figure 3B), but the latter involved upper theta (6–8 Hz) phase (Figure 3B). Of the six patients who carried out the novelty oddball task, Patients 2, 3 and 5 had significant left ATN PAC during performance of the oddball task (Figure 3C), but a narrower HFB range was involved. The average theta–gamma PAC (LFO: 4–6 Hz; HFB 83–148 Hz) was greater during resting than during the externally-focused tasks (Wilcoxon test: *p* = 0.0018). This PAC was also greater during rest than during each externally-focused task separately (rest vs. encoding, Wilcoxon test: *p* = 0.0082); rest vs. oddball, Wilcoxon test: *p* = 0.047). On direct comparison of the frequency ranges involved, PAC differed between rest and both cognitive tasks (rest vs. encoding: 2-D KS test: K-statistic = 0.49, *p* = 0.0015; rest vs. novelty oddball: K-statistic = 0.43, *p* = 0.054; Figure 4). Also of note is that theta–gamma PAC during the memory-based task involved a lower gamma band, as previously reported (Sweeney-Reed et al., 2014). A greater number of frequency combinations showed significant PAC during rest than during the tasks involving an external focus of attention (Wilcoxon test: *p* = 0.0025). The finding was not altered when comparing rest with the encoding task alone (Wilcoxon test: *p* = 0.0082) or with the oddball task alone (Wilcoxon test: *p* = 0.022). The number of significant frequency combinations did not differ between the two externally-focused tasks (Wilcoxon test: *p* = 0.28).

**Figure 5:**
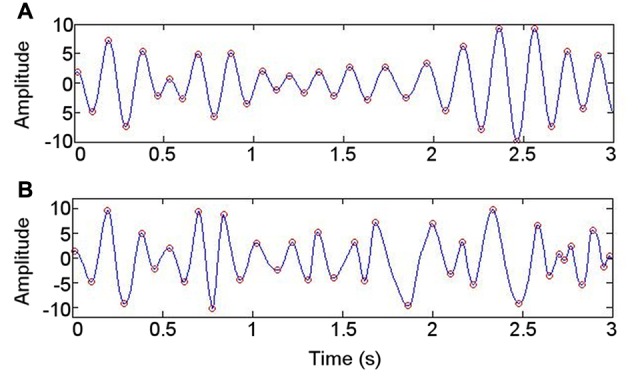
Illustration of approach to evaluating whether data are nonsinusoidal. **(A)** Bandpass filtering at 4–6 Hz. **(B)** Intrinsic mode function with spectral peak in 4–6 Hz range, derived using empirical mode decomposition.

The waveform sharpness ratio calculated from the IMF with greatest power in the 4–6 Hz range correlated with that derived from the 4–6 Hz band obtained using a bandpass filter (*r* = 0.84; *p* = 0.019; Figure 5). As might be expected from a data-driven decomposition, the sharpness ratio was higher for each participant when derived using EMD (paired *T*-test: *T* = −3.02; *p* = 0.024). No correlation was detected between the sharpness ratio (derived using either approach) and either the mean PAC or the highest PAC in the frequency ranges at which PAC was present in each participant at rest (4–6 Hz phase coupled with 80–150 Hz amplitude; *p* > 0.05).

**Figure 6:**
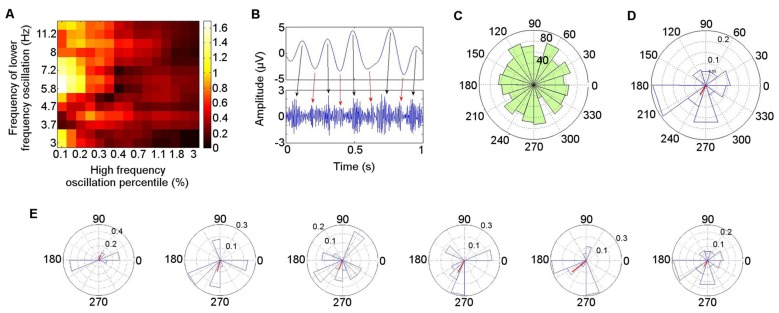
Detailed illustration of phase–amplitude coupling (PAC) during rest in Patient 4. **(A)** Low frequency oscillatory range at which coupling with high frequency band (80–150 Hz) amplitude was greatest. **(B)** Coupling between theta (4–6 Hz) phase and gamma (80–150 Hz) amplitude in bandpass filtered signals. **(C)** Polar representation of PAC calculated over 30 s pooled across all seven participants. The theta phase is shown radially, with mean resultant vector length (red) representing consistency of the phase over windows with the highest gamma amplitude (Miyakoshi et al., 2013). **(D)** PAC over 30 s in Patient 4. **(E)** PAC in Patient 4, in 5 s time windows.

The PAC in the left ATN during the resting condition is illustrated in detail for Patient 4 (Figure 6), who is representative of the group. A Hamming windowed FIR filter was applied to the data to extract the 80–150 Hz band, from which amplitudes were derived. The 80–150 Hz band was selected on the basis of our finding that activity in this range was coupled with theta oscillatory phase both during rest in each participant in the left ATN, and also in previously reported neocortical theta–HFB PAC (Canolty et al., 2006). Coupling with the phase of low frequency oscillations ranging from 3–12 Hz was calculated, to identify the low frequency range most strongly coupled with the gamma amplitudes, computing all possible frequency combinations between the low and the high frequency oscillations, using an increasing percentage of the highest absolute value points from the high frequency oscillation (Miyakoshi et al., 2013; Figure 6A). The MI between the phases of the 4–6 Hz oscillation and the amplitudes of the 80–150 Hz oscillation was significant (permutation test: *p* = 0.0067; Miyakoshi et al., 2013). PAC can be observed between the raw theta and gamma bands (Figure 6B). Note that during the arbitrarily selected 1 s of data, while the strongest coupling takes place between theta phase peaks and gamma amplitude peaks (black arrows), smaller gamma amplitude peaks (red arrows) are also coupled with the theta phase troughs.

The distribution of the mean theta (4–6 Hz) phase angle at which HFB (80–150 Hz) oscillations peaked during rest varied across participants, and when these phase angles were pooled across participants, their distribution did not differ from a normal circular distribution (Kuiper’s test: *p* > 0.05), reflecting the inter-participant differences in preferred theta phase (Figure 6C). When each participant was taken individually, however, the theta phase distribution at which HFB power peaked differed from a normal circular distribution (Kuiper’s test: *p* < 0.005). Directly comparing the theta phase distributions from individual participants pairwise between patients, the distribution of the theta phase at which HFB peaked differed between participants in 10 of the 21 (48%) pairwise comparisons (two-sample Kuiper test: *p* < 0.0024—Bonferroni corrected threshold for criterion *q* = 0.05). A qualitative analysis was performed on the first 30 s of clean data from each participant to examine whether the preferred LFO phase varied over time. A window length of 5 s was applied, selected with reference to measurement of lengths of episodes of spontaneous mind-wandering ranging between 2.5 s and 10 s (Henríquez et al., 2016) and between 4 s and 5 s (Smallwood, 2013), to provide an indication of the variability of the phase at which HFB amplitude peaked for each participant. This revealed that the theta phase angle at which HFB peaked in any individual showed consistency over time. The mean angle in each window varied less than 45° in at least four of the six windows in every participant. The finding is illustrated for Participant 4 (Figure 6D). The mean theta distribution at which HFB power peaked over the entire 30 s epoch was 242°, with four out of six of the 5 s windows showing a HFB peak at a theta phase angle within 22° of the mean angle for the 30 s interval (67°, 255°, 248°, 242°, 220°, 186°; Figure 6E). The theta phase angle at which HFB power peaked was within a range of 35° in three out of six windows in six of the seven participants and within 60° in one participant.

Event-related theta–gamma coupling has previously been reported during the memory encoding task, but between theta (5–6 Hz) phase and low frequency gamma (40–50 Hz) power (Sweeney-Reed et al., 2014). We also examined coupling in the continuous encoding data, involving these frequencies as well as the frequencies identified here as coupled during rest (theta: 4–6 Hz; HFB: 80–150 Hz). While the theta phase distribution coupled with low gamma power differed from a normal distribution across participants (Kuiper’s test: *p* < 0.001), the theta phase angle distribution coupled with HFB power did not differ from a normal distribution, either across participants or in any individual participant. Moreover, the theta phase distribution at which gamma power peaked at rest differed significantly from that during encoding when directly compared (Kuiper’s test: *p* < 0.001), both examining theta phase coupled with the HFB and also theta phase coupled with low frequency gamma activity.

## Discussion

We show that theta–HFB PAC is present in the ATN during the resting state, with HFB activity peaking at varying phases in the theta cycle across participants. PAC between theta (4–6 Hz) oscillations and HFB (80–150 Hz) activity was greater during rest than during tasks with an external focus of attention and involved a broader HFB frequency range. PAC is thought to provide a means of communication between distinct neuronal populations (Canolty et al., 2006; Axmacher et al., 2010; Canolty and Knight, 2010), as well as a mechanism by which particular neural populations may be selected (Yanagisawa et al., 2012; Sweeney-Reed et al., 2014). On this basis, we consider a possible role for this coupling in the thalamus in task-switching. We then discuss the implications of the frequency ranges involved, individual variation, and the possibility of lateralization.

We identified greater theta–HFB PAC during rest than during the tasks involving an external focus of attention, and broader gamma frequency range coupling with the theta rhythm. The involvement of a broader ATN gamma frequency range during rest is consistent with diverse cortical regions projecting to the ATN (Aggleton et al., 2010) and with an engagement of the ATN in multiple processes when the mind is wandering. ECoG measurements reveal activity from local neocortical neuronal populations, and differing spatial patterns of theta–HFB PAC have been reported during different cognitive tasks (Canolty et al., 2006). Driving of PAC in the ATN during an internally-focused state may be linked to PAC occurring in widespread neocortical regions, involving a correspondingly broad gamma frequency range. In contrast, during a specific cognitive task, fewer neocortical populations may be engaged. Indeed, we previously reported activity in a narrower gamma frequency range to be coupled with theta oscillations during successful compared with unsuccessful memory formation and suggested that the PAC reflected a process in which stimulus-relevant gamma-firing populations were selected through entrainment by theta oscillations (Sweeney-Reed et al., 2014). The reduced PAC and more focused gamma frequency band engaged in PAC are consistent with a role for the ATN in switching between cognitive states. Known anatomical and functional connectivity between the ATN and neocortex (Aggleton et al., 2010; Fitzgerald et al., 2013; Sweeney-Reed et al., 2014) places the ATN in a position to play a role in switching between cognitive states and tasks, and differing thalamic PAC in internally compared with externally focused attentional states provides a potential mechanism by which local activity relevant to an external task is selected and coordinated.

A spectral power peak in the theta frequency range involved in PAC at rest (4–6 Hz) was observed, which is consistent with reports that the theta oscillation dominates in the ATN (Vertes et al., 2001). A peak in the LFO is a prerequisite for PAC (Aru et al., 2015) and is usually observed when theta–gamma PAC is detected (Axmacher et al., 2010; Foster and Parvizi, 2012; Scheffer-Teixeira et al., 2012). There was no clear peak in the HFB range. Accordingly, coupling between bursts of HFB activity and the theta rhythm is not contingent on an overall peak in HFB in the power spectrum. PAC reflects the timing of gamma bursts rather than their amplitude over the entire observed time period. Neocortical PAC detected during rest in MEG and in ECoG recordings was also not associated with a corresponding gamma spectral peak (Foster and Parvizi, 2012; Roux et al., 2013). Similarly, in task-related hippocampal recordings, theta–gamma PAC was detected without a corresponding gamma peak in the power spectrum (Axmacher et al., 2010; Scheffer-Teixeira et al., 2012). In the latter two studies, the authors highlighted that these gamma peaks can only be detected when examining particular phases of the theta cycle, underlining the importance of PAC measurement in investigations of the role of high frequency activity.

The distribution of the theta and broadband HFB frequencies involved in significant PAC was consistent across patients during the resting state, but not during the tasks involving an external focus of attention. In previous studies reporting PAC in the ATN, PAC was calculated over short (1 s) post-stimulus epochs, in which a particular aspect of cognitive processing (memory formation) was present in each epoch (Sweeney-Reed et al., 2014, 2015). The task-relevant PAC was apparent through the averaging across these epochs. In the current study, PAC was calculated over longer, continuous data, during which the cognitive processing was unconstrained (resting state) or constrained (viewing photographic scenes). Our finding that PAC encompassed a broader gamma frequency range during rest fits with the idea that multiple, diverse (in terms of the relevant gamma frequency range) neural assemblies form and dissolve over time when attention is internally focused. By contrast, during a specific task, only the task-relevant assemblies would continue to be coordinated by the timing of theta oscillations. In other words, a repeated task would be expected consistently to activate the same task-relevant brain networks, which would not be expected in an unconstrained state.

The PAC identified here involves the theta and HFB frequency ranges. Localized gamma activity has been observed to depend on stimulus features, but conduction delays in neuronal signal transmission through the cortex render long-range coordination between high frequency gamma rhythms improbable (Ray and Maunsell, 2015). Our findings are consistent with the suggestion that long-range communication involves the theta frequency range, whose timing coordinates local object representations encoded in the HFB range. In the case of resting state activity, such object representations involve internal attentional foci. It should be noted, however, that long-range synchrony in lower frequency gamma bands has recently been observed between the inferior frontal junction and the parahippocampal place (60–90 Hz) and fusiform face (70–100 Hz) areas during an object-based attention task using MEG (Baldauf and Desimone, 2014).

The present findings at rest pertain to thalamic HFB activity. The specific roles of coupling involving high compared with low gamma frequency activity require further investigation. Human ECoG recordings over the left frontotemporal cortex have revealed differing patterns of theta–gamma PAC during processing of different cognitive tasks involving HFB activity (Canolty et al., 2006). Coupling between theta phase and HFB amplitude has also been identified during rest in ECoG data (Foster and Parvizi, 2012). During memory processing, low gamma oscillations have been found to be coupled with the theta rhythm in the neocortex (ECoG), in the hippocampus, and also in the ATN (Tort et al., 2009; Axmacher et al., 2010; Li et al., 2012; Fitzgerald et al., 2013; Sweeney-Reed et al., 2014). In line with the latter findings, low rather than high frequency gamma power was coupled with theta oscillatory phase during memory encoding in the present study, despite averaging over multiple trials including both successful and unsuccessful memory formation. ATN PAC between LFOs and low frequency gamma (30–50 Hz) activity has previously been reported in a study investigating PAC involving gamma frequencies up to 64 Hz in one patient at rest and in another patient during a memory task (Fitzgerald et al., 2013). The current study expands on this work, by investigating PAC involving higher frequency gamma activity (the HFB range) and assessing the consistency of the frequency ranges involved in PAC across multiple participants. We compare in detail our findings with those of Fitzgerald et al. (2013), for the lower gamma frequencies included in both studies. Although theta–gamma PAC is commonly observed, PAC involving other frequency ranges is being increasingly reported (Maris et al., 2011; Van der Meij et al., 2012). Fitzgerald et al. (2013) identified PAC between upper theta/alpha phase and low frequency gamma activity during memory task performance. Four of our patients also showed PAC during the memory task involving theta and lower gamma frequencies, but the frequency of the LFO coupled with lower frequency gamma was only as high as that reported by Fitzgerald et al. (2013) in Patient 5. Differences may result from differences in the paradigms performed. During rest, Fitzgerald et al. (2013) observed PAC in a patient between alpha/beta phase and low frequency gamma activity. While three of our seven patients did also show significant PAC between low frequency gamma activity and alpha or beta phase (four of seven patients), these findings were less consistent across patients than those involving lower theta phase and HFB (all seven patients).

We observed that the preferred theta phase, at which the amplitude of gamma activity was greatest, varied across participants during rest. Increased gamma band amplitude has been found at specific times in the theta cycle (Canolty and Knight, 2010). Specifically, gamma power increases have been observed at the theta phase troughs in ECoG data recorded during auditory processing (Canolty et al., 2006), at theta peaks in the hippocampus during working memory processing (Axmacher et al., 2010) and in the ATN (Sweeney-Reed et al., 2014) and hippocampus (Lega et al., 2016) during memory encoding, and at both theta peaks and troughs in the electroencephalogram during visual processing (Jacobs and Kahana, 2009). During memory retrieval, the phase of beta oscillation at which gamma power increased differed between hits and correct rejections (Staudigl et al., 2012). In the present study, coupling was evaluated over a continuous time period, rather than on an event-related basis. We suggest that the inter-participant variability in preferred theta phase at rest reflected inter-individual variation in cognitive processing when no specific task was performed. Indeed, although specific brain regions have been consistently identified as being active during the resting state (Fox et al., 2006; Vincent et al., 2006), the processes that are engaged would be expected to differ between participants when not performing a specified cognitive task, since the brain’s state is influenced by modulatory factors such as mood, arousal, and fatigue. Given individual differences in cognition at rest, the variation of the theta phase at which gamma oscillatory amplitude peaks was to be expected. It is noteworthy that the theta phase at which gamma power peaked showed some consistency over a 30 s time period within individual participants. We tentatively suggest that this finding could reflect particular internal attentional foci during the observed time period in individual participants.

Investigation of PAC in neural data has proliferated in the last decade, but concerns have been posed that PAC detection may be spurious under certain circumstances (Aru et al., 2015; van Ede et al., 2015; Jensen et al., 2016; Cole and Voytek, 2017; Vaz et al., 2017). Certain features in the data have been proposed to be important in interpreting PAC (Aru et al., 2015; Jensen et al., 2016). The power of the LFO in the frequency range for which PAC was greatest at rest was lower during rest than during the memory task, for which less PAC was identified (Figure 2). This finding supports the notion that the PAC detected has a neural basis (Aru et al., 2015; Jensen et al., 2016). Furthermore, the detection of gamma activity (visible in the data in Figure 5B) means that the PAC is unlikely to result from high frequency harmonics rather than high frequency activity (Jensen et al., 2016).

PAC has been proposed as a mechanism by which information is integrated across multiple spatiotemporal scales (Canolty and Knight, 2010). Recent findings of a strong correlation between the sharpness (a quantification of the extent to which the waveform is sawtooth in shape) of motor cortical beta oscillations and PAC have led to the suggestion that PAC could represent another phenomenon in such cases, possibly reflecting synaptic input synchrony (Cole et al., 2017). The thalamic theta LFO involved in PAC here did not have the marked sawtoothed shape observed in the motor cortical beta oscillations investigated by Cole et al. (2017). Moreover, the theta–HFB PAC in the resting thalamic recordings did not correlate with the sharpness ratio of the LFO, which is consistent with our interpretation that resting thalamic PAC could reflect information integration.

Our consistent finding of theta–HFB PAC only in the left ATN for each individual suggests a possible lateralization of function in the role of the ATN, although we note that the difference between PAC levels in the left ATN and right ATN was not significant on direct comparison. Asymmetry in thalamic functional connectivity in resting state fMRI has been previously reported (Saenger et al., 2012). Furthermore, differences in left and right thalamic activity in different visual discrimination and memory tasks have been observed in positron emission tomography studies (Shulman et al., 1997), and the suggestion of lateralization also fits with the left thalamus having previously been identified as exhibiting different cognitive correlates to the right thalamus during rest in an fMRI study in which resting left but not right thalamic regional homogeneity correlated with a happiness index, which could reflect either personality or a persistent emotional state (Luo et al., 2014). Greater functional connectivity on the left but not on the right side has been identified in the prefrontal–anterior thalamic (involving the ventral anterior nucleus) network in patients with idiopathic generalized epilepsy compared with controls in a resting state fMRI study of corticothalamic networks (Ji et al., 2015). The authors do not speculate regarding this laterality, but we note that their patients were all right-handed. All participants except Patient 5 in the current cohort are also right-handed. However, theta–HFB coupling in Patient 5 was identified in both the left and the right ATN. Patient 2 also showed theta–HFB PAC in the right ATN, and this patient is right-handed. These findings do not support handedness as an explanation of the left ATN results. Power was found to be greater in the ipsilateral than contralateral ATN on resting state fMRI in patients with unilateral TLE (Morgan et al., 2016). Five of our patients had bilateral epileptic foci (including Patient 2), so that our findings also do not correspond with seizure zone laterality. Of further note is that our previous finding of theta phase synchrony and theta–gamma PAC during encoding of visual stimuli involving the ATN was significant only for the right ATN (Sweeney-Reed et al., 2014). Further studies are required to elucidate the possibility of differing roles for the left and right ATN during the resting state. Despite a memory-related role for both the ATN and the DMTN, anatomical studies show that they have different connectivity (Aggleton, 2012), and lesion and imaging studies dissociate their roles during task performance (Pergola et al., 2013; Wolff et al., 2015; Wright et al., 2015; Leszczynski and Staudigl, 2016), which could explain the differing findings for the different nuclei. Specifically, the ATN is thought to play a role in recollection, while the DMTN is a part of a network supporting familiarity (Aggleton et al., 2010; Aggleton, 2012). The ATN have also been postulated to be involved in selection of information for storage, while the DMTN may coordinate and select retrieval strategies (van der Werf et al., 2003). Further studies are required to investigate the extent to which PAC plays a role in the separate corticothalamic networks in which each nucleus is involved.

In conclusion, we have identified consistent coupling between theta (4–6 Hz) oscillatory phase and the amplitude of HFB (80–150 Hz) activity in the ATN during rest, which was absent during tasks involving an external focus of attention. These findings support the notion that PAC represents ongoing processes in subcortical as well as cortical brain structures, which are modulated during particular cognitive acts. Resting thalamic PAC may reflect a state of readiness for activation of networks involved in specific cognitive tasks.

## Author Contributions

CMS-R: study conception; CMS-R, MDR and RTK: study design; TZ and JV: data acquisition; CMS-R: electrophysiological data analysis and interpretation; JV, LB, FCS and CMS-R: electrode localization and evaluation; CMS-R: drafting of manuscript; TZ, JV, FCS, LB, VB, MW, HH, H-JH, MDR and RTK: data interpretation and manuscript revision. All authors read and approved the final manuscript.

## Conflict of Interest Statement

The authors declare that the research was conducted in the absence of any commercial or financial relationships that could be construed as a potential conflict of interest.
