# Supplementary material for: Anterior Thalamic High Frequency Band Activity Is Coupled with Theta Oscillations at Rest
Source: Front Hum Neurosci. 2017 Jul 20;11:358. doi: 10.3389/fnhum.2017.00358 (PMC5518534; doi:10.3389/fnhum.2017.00358)
Supplement: Supplementary file 1 [file Data_Sheet_1.docx]

Supplementary Material

Anterior Thalamic High Frequency Band Activity is Coupled with Theta Oscillations at Rest

**Catherine M. Sweeney-Reed^1*^, Tino Zaehle^1^, Jürgen Voges^1,2^, Friedhelm C. Schmitt^1^,**

**Lars Buentjen^1^, Viola Borchardt^2^, Martin Walter^2,4^, Hermann Hinrichs^1,2,3^, Hans-Jochen Heinze^1,2,3^, Michael D. Rugg^5^, Robert T. Knight^6^**

*** Correspondence:** Catherine M. Sweeney-Reed: catherine.sweeney-reed@med.ovgu.de

# Supplementary Figures


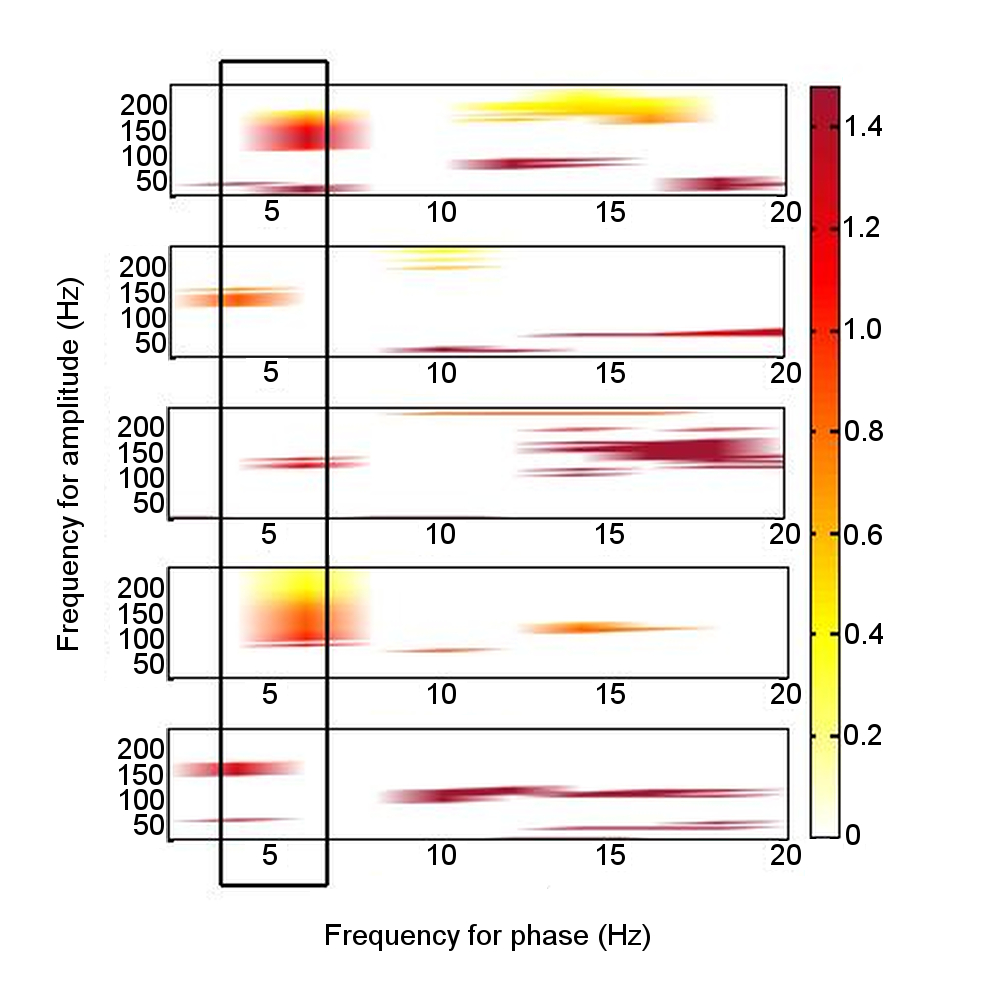


**Supplementary Figure 1.** **Phase**–**amplitude coupling (PAC) at rest between theta phase and high frequency band amplitude in the left anterior thalamic nucleus in additional clean data.** PAC is shown when it was significant (permutation test: criterion p < 0.05). The analysis was performed with additional clean data where available. The top shows data from Patient 2, the middle three panels from Patient 3, and the bottom panel from Patient 5.
